# Reactive intermediates in copanlisib metabolism identified by LC-MS/MS: phase I metabolic profiling†

**DOI:** 10.1039/c8ra10322d

**Published:** 2019-02-21

**Authors:** Haitham AlRabiah, Adnan A. Kadi, Mohamed W. Attwa, Ali S. Abdelhameed, Gamal A. E. Mostafa

**Affiliations:** Department of Pharmaceutical Chemistry, College of Pharmacy, King Saud University P. O. Box 2457 Riyadh 11451 Saudi Arabia mzeidan@ksu.edu.sa +966 1146 76 220 +966 1146 70237; Students' University Hospital, Mansoura University Mansoura 35516 Egypt; Micro-analytical Lab, Applied Organic Chemistry Department, National Research Center Dokki Cairo Egypt

## Abstract

Copanlisib (CNB; Aliqopa™) is a novel, intravenous phosphoinositide 3-kinase inhibitor used to treat various solid and hematological malignancies. CNB was recently approved by the U.S. FDA to treat adults that relapsed after two preceding systemic therapies. Using LC-MS/MS, we screened for the *in vitro* metabolites of CNB formed in human liver microsomes (HLMs) and probed for the generation of reactive electrophiles using methoxyamine and potassium cyanide as nucleophiles to capture reactive electrophiles by forming stable adducts that are suitable for identification by LC-MS/MS. Seven CNB phase I metabolites generated by oxidation, hydroxylation, oxidative dealkylation, reduction, and *N*-oxidation were identified. In addition, four reactive electrophiles, 2 aldehydes and 2 iminium ions, were identified, and a prediction of the corresponding bioactivation mechanism is presented. The formation of reactive metabolites may be associated with the side effects reported for CNB. To our knowledge, this is the first report on the detailed structural characterization of reactive intermediates generated in CNB metabolism.

## Abstract 2

Copanlisib (CNB; Aliqopa™) is a novel, intravenous phosphoinositide 3-kinase inhibitor used to treat various solid and hematological malignancies.

## Graphical abstract

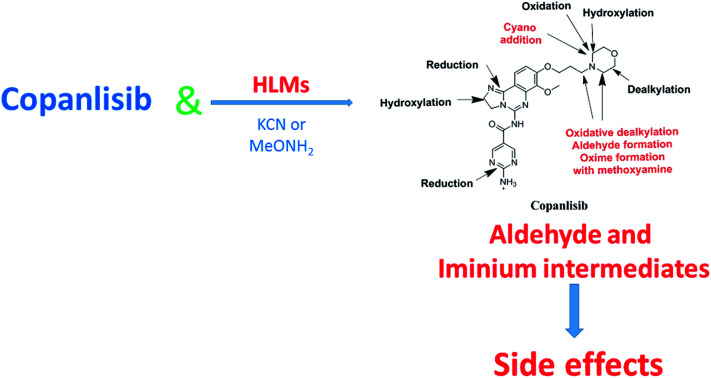

## 1. Introduction

Phosphatidylinositol 3-kinase (PI3K) is an important target in the clinical management of different types of cancer as its biological activity is fundamental to interpreting how extracellular stimulation translates into intracellular signaling reactions, including cell survival and growth. The overexpression of PI3K isoforms is associated with a poor prognosis and considered as a major cause of relapse and cancer resistance in B-cell malignancies such as follicular lymphoma.^1–3^ Gene mutation encoding phosphatase and tensin homolog (PTEN) and PI3K are responsible for reversing PI3K phosphorylation and are considered to be among the most commonly observed solid tumor alterations.^3–5^ Therefore, there are many promising candidate PI3K inhibitor drugs currently under clinical evaluation for the treatment of a variety of blood cancers and solid tumors.^3,6,7^ Idelalisib, a PI3Kδ-selective inhibitor, has been approved for indolent non-Hodgkin's lymphoma treatment^8^ but has no clinical benefit in solid tumors.

Copanlisib (CNB) is a potent class I PI3K inhibitor with preferential activity against p110α and p110δ than p110β and p110γ. CNB is a novel, intravenous PI3K inhibitor used to treat different solid and hematological malignancies.^9,10^ CNB induces tumor cell death such as apoptosis, and inhibits primary malignant B cell proliferation and tumor growth in the preclinical xenograft tumor models of malignant B cell.^11^

CNB (Aliqopa™) is approved by the U.S. Food and Drug Administration for the treatment of adults who relapsed after two prior treatments with systemic therapies.^12^ Common toxic side effects of CNB include weakness, hypertension, hyperglycemia, diarrhea, nausea, low levels of specific white blood cells (leukopenia, neutropenia), low levels of blood platelets, and lower respiratory tract infections.^13^

Metabolic detoxification involves pathways that transform endogenous compounds and xenobiotics into more hydrophilic species to facilitate excretion from the human body. The generated metabolites are often less toxic than the parent molecules but in some cases, bioactivation may occur and promote reactive electrophile formation which leads to more toxic metabolites.^14–16^ Reactive electrophiles are electron deficient and can modify proteins and DNA by forming covalent bonds; this is considered the first step in drug-mediated organ toxicities.^17,18^ Verifying reactive metabolite production is a critical task in the study of drug-induced toxicity. Reactive metabolites are often formed by phase-I metabolic reactions and cannot be directly characterized because of their transient nature. Instead, a trapping agent can be used to capture reactive intermediates *via* the formation of stable adducts that can be identified by mass spectrometry.^19,20^

**Fig. 1:**
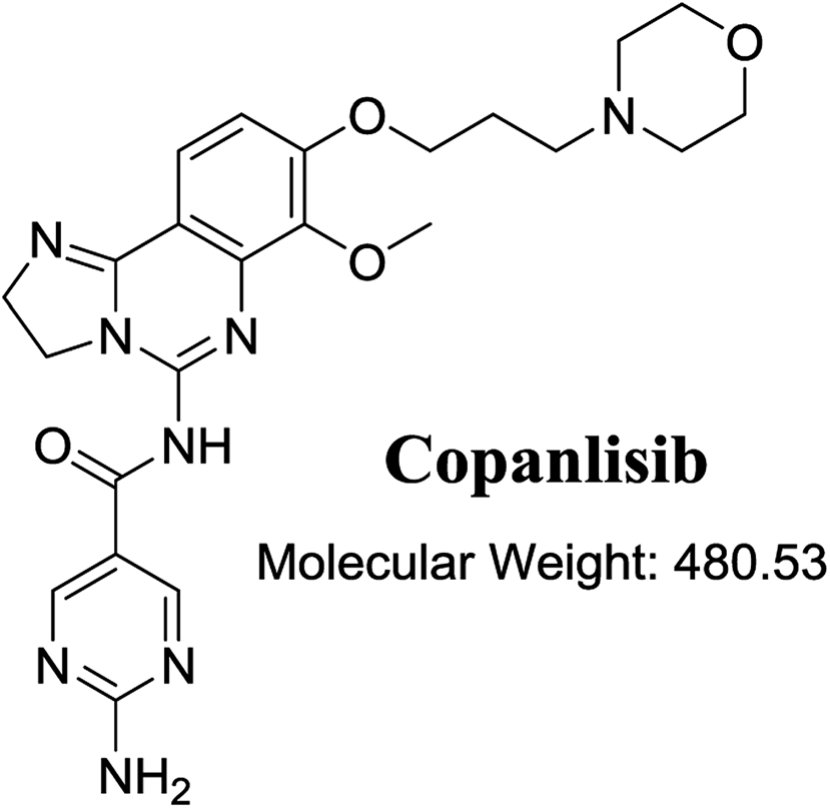
Chemical structure of copanlisib.

The structure of CNB, 2-amino-*N*-{7-methoxy-8-[3-(4 morpholinyl)propoxy]-2,3-dihydroimidazo[1,2-*c*]quinazolin-5-yl}-5-pyrimidinecarboxamide (Fig. 1), contains a morpholine moiety (cyclic tertiary amine ring) that can undergo bioactivation by iminium ion generation or oxidative dealkylation to form aldehyde intermediates.^21–24^ Glutathione and its derivatives are highly nucleophilic and react poorly with strong electrophiles.^25^ However, the iminium ion and aldehydes are electrophiles that can be trapped using potassium cyanide and methoxyamine, respectively.^14,21,22^ The adducts formed by nucleophilic–electrophilic interactions are considered stable and can be separated and identified by LC-MS/MS.^19–21,26,27^ We hypothesized that these reactive intermediates are potentially involved in the reported side effects of CNB.

Literature review showed a single article that described the pharmacokinetics and disposition of CNB in human volunteers,^28^ without addressing the structural identification of the formed metabolites or reactive intermediate formation. Therefore, the aim of the current work is to identify the bioactivation pathways of CNB resulting in reactive intermediates as can only be captured *via in vitro* experiments. While *in vivo*, these compounds have the capability to bind covalently to DNA and protein molecules and become undetectable.^17,20,26,29^

## 2. Chemicals and methods

### 2.1. Chemicals

All chemical and solvents used were of analytical grade. CNB was procured from Med Chem. Express (USA). Acetonitrile, methoxyamine, ammonium formate, pooled human liver microsomes (HLMs, M0567) potassium cyanide and formic acid were procured from Sigma-Aldrich (USA). HPLC grade water (H_2_O) was purchased from Milli-Q plus system (USA).

### 2.2. Chromatographic conditions

Parameters used for chromatographic separation of the HLM incubation mixture are shown in Table 1.

**Table 1:** LC-MS/MS optimized parameters

LC			MS/MS	
LC	Agilent 1200		MS	Agilent 6410 QqQ
Gradient mobile phase	A: 10 mM ammonium formate in H_2_O		ESI	Positive ESI
	B: acetonitrile			Drying nitrogen gas, 12 L min^−1^ flow rate, 60 psi pressure
	0.5 mL min^−1^ flow rate			
	65 min run time			
C_18_ column (Agilent eclipse plus)	250 mm length			350 °C
	4.6 mm ID			4000 V capillary voltage
	5 μm particle size		Collision gas	High purity nitrogen gas
	23 ± 2 °C		Modes	MS scan and PI
Gradient mobile phase	Time	% acetonitrile	Drug	CNB and its related metabolites
	0	5		
	5	5		
	40	60		
	60	90		
	65	5		
			Analyzer	145 eV fragmentor voltage
				25 eV collision energy

### 2.3. HLM incubation

Several concentrations from 5 to 30 μM were analyzed; however, the only change observed was an increase in the concentration of metabolites to allow easier identification. Screening for *in vitro* metabolites of CNB was performed by incubating 30 μM CNB with 1.0 mg mL^−1^ HLMs in phosphate buffer (50 mM, pH 7.4) and 3.3 mM MgCl_2_. Incubation time and temperature were 2 h and 37 °C, respectively. The samples were incubated in a shaking water bath. The CNB metabolic reactions were initiated by adding NADPH (1.0 mM) and stopped by adding 2 mL ice-cold acetonitrile. Protein precipitates were removed by centrifugation at 9000*g* (15 min, 4 °C) and the supernatants were evaporated then reconstituted in the mobile phase. Aliquots of 10 μL of each reconstituted sample were analyzed using LC-MS/MS system.^30–32^ Replicates of blank sample were analyzed at the same run to confirm the absence of the proposed metabolites or adducts (ESI Fig. S11–S20†).

### 2.4. Identification of CNB reactive metabolites

Full MS scans and extracted ion chromatograms of select *m*/*z* peaks were used to identify *in vitro* metabolites from the incubation chromatograms; molecular ions served as precursor ions (PIs) for fragmentation into daughter ions (DIs). Fragmentation (F) patterns were used to characterize *in vitro* metabolites and reactive intermediates generated by CNB metabolism.

The same HLM incubation assay was repeated in the presence of methoxyamine or potassium cyanide to trap bioactive electrophiles. Reactions were performed in triplicate.

## 3. Results and discussion

### 3.1. Fragmentation analysis of CNB

**Fig. 2:**
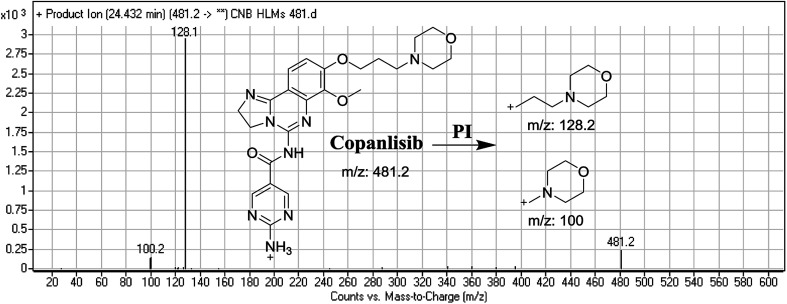
Mass spectrum of copanlisib and its MS/MS fragments.

The CNB precursor ion peak (PIP) appeared at 24.4 min. F of PI at *m*/*z* 481 resulted in two DRs at *m*/*z* 128 and *m*/*z* 100, representing alkyl morpholine rings (Fig. 2).

### 3.2. Identification of CNB *in vitro* metabolites and reactive intermediates

Purified extracts recovered from HLM assays were subjected to LC triple-quadrupole MS (LC-QqQ MS) from which, three new phase I metabolites (M499, M483a, and M497a) and 4 reactive metabolites were identified. Six metabolites were produced from seven phase I metabolic reactions namely, (α-hydroxylation, α-oxidation, reduction, oxidative dealkylation, and *N*-oxidation. In addition, two cyano and 2 methoxyamine adducts were identified (Table 2).

**Table 2:** Phase I and reactive metabolites of copanlisib

	MS scan	Most abundant fragment ions	Retention time (min)	Metabolic reaction
CNB	481	128, 100	24.4	

**Phase I metabolites**				
M497a	497	479, 358, 128	23.4	Hydroxylation of 2,3-dihydroimidazole ring
M497b	497	479, 144, 126	23.8	α-Hydroxylation at morpholine ring
M495	495	142, 233, 354	28.0	α-Oxidation
M455	455	102, 233, 354	23.0	Ring cleavage and dealkylation
M483a	483	100, 128, 361	24.1	Reduction at 2,3-dihydroimidazole
M483b	483	130, 233, 354	26.1	Morpholine ring cleavage, oxidative dealkylation.
M499	499	100, 128, 361	22.7	Reduction of dihydroimidazole ring & *N*-oxidation of pyrimidine ring

**Reactive metabolites**				
M506	506	479, 354, 126, 98	32.6	Cyano addition
M508	508	481, 356, 260, 126, 98	32.7	Reduction and cyano addition
M439	439	354, 122, 94	51.5	Methoxyamine oximer formation
M512	512	354, 272, 131	37.2	Oxidative dealkylation of morpholine ring then methoxyamine oximer formation

#### 3.2.1. Identification of M497a and M497b phase I metabolite of CNB

**Fig. 3:**
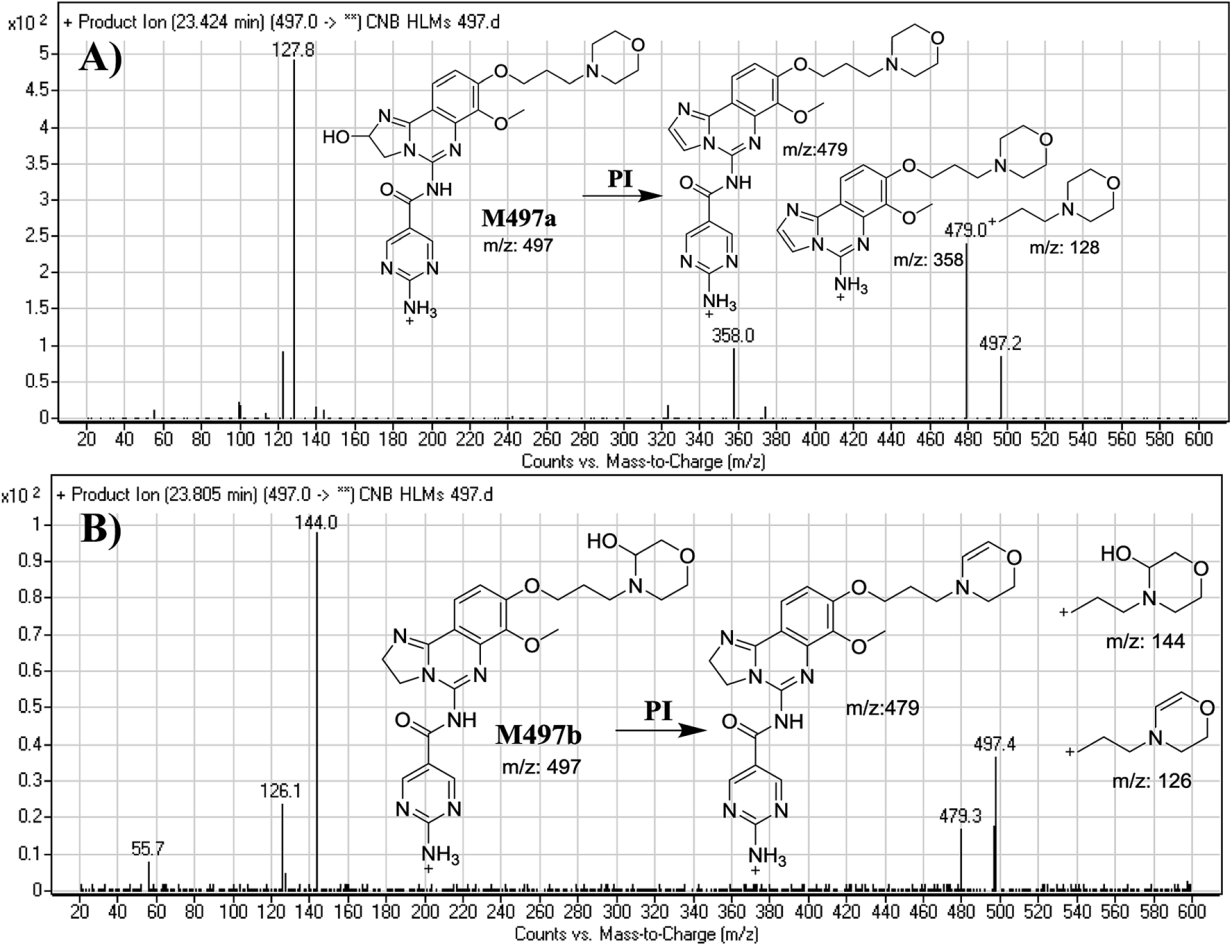
Mass spectra of M497a (A) and M497b (B), and their MS/MS fragments.

M497a and M497b PIPs appeared at 23.4 min and 23.8 min, respectively. F of PI at *m*/*z* 497 produced different DRs (Fig. 3A and B).

The F of M497a resulted in three DRs at *m*/*z* 479, *m*/*z* 358, and *m*/*z* 128 (Fig. 3A), all containing the alkyl morpholine ring. The DR at *m*/*z* 128 suggested the absence of a metabolic change in the morpholine moiety. DRs revealed that M497a is a hydroxylated form of CNB with a 2,3-dihydroimidazole ring matching the DR at *m*/*z* 358 (Fig. 3A).

The F of M497b resulted in three DRs at *m*/*z* 479, *m*/*z* 144, and *m*/*z* 126 (Fig. 3B). The DR at *m*/*z* 142 represented the alkyl hydroxyl morpholine ring produced by single bond cleavage which matches the DR at *m*/*z* 126. Hydroxylation was predicted to occur in the α-position of the morpholine nitrogen (N) atom (Fig. 3B).

#### 3.2.2. Identification of M495 phase I metabolite of CNB

**Fig. 4:**
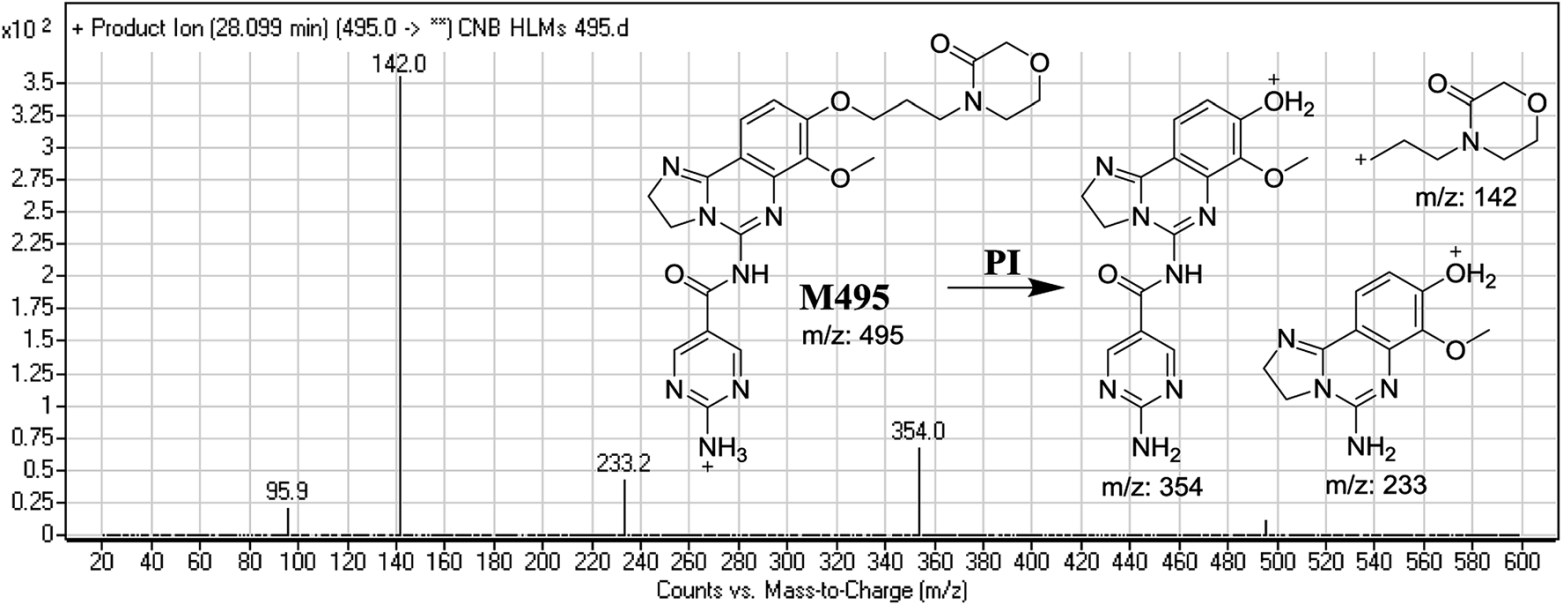
Mass spectrum of M495 and its MS/MS fragments.

M495 PIP appeared at 28.0 min. F of PI at *m*/*z* 495 resulted in three DRs at, *m*/*z* 354, and *m*/*z* 233, *m*/*z* 142. DR at *m*/*z* 142 represented an alkyl keto morpholine ring matching the other ions at *m*/*z* 354 and *m*/*z* 233. Oxidation was predicted to occur at the α-position of the morpholine N atom (Fig. 4).

#### 3.2.3. Identification of M455 phase I metabolite of CNB

**Fig. 5:**
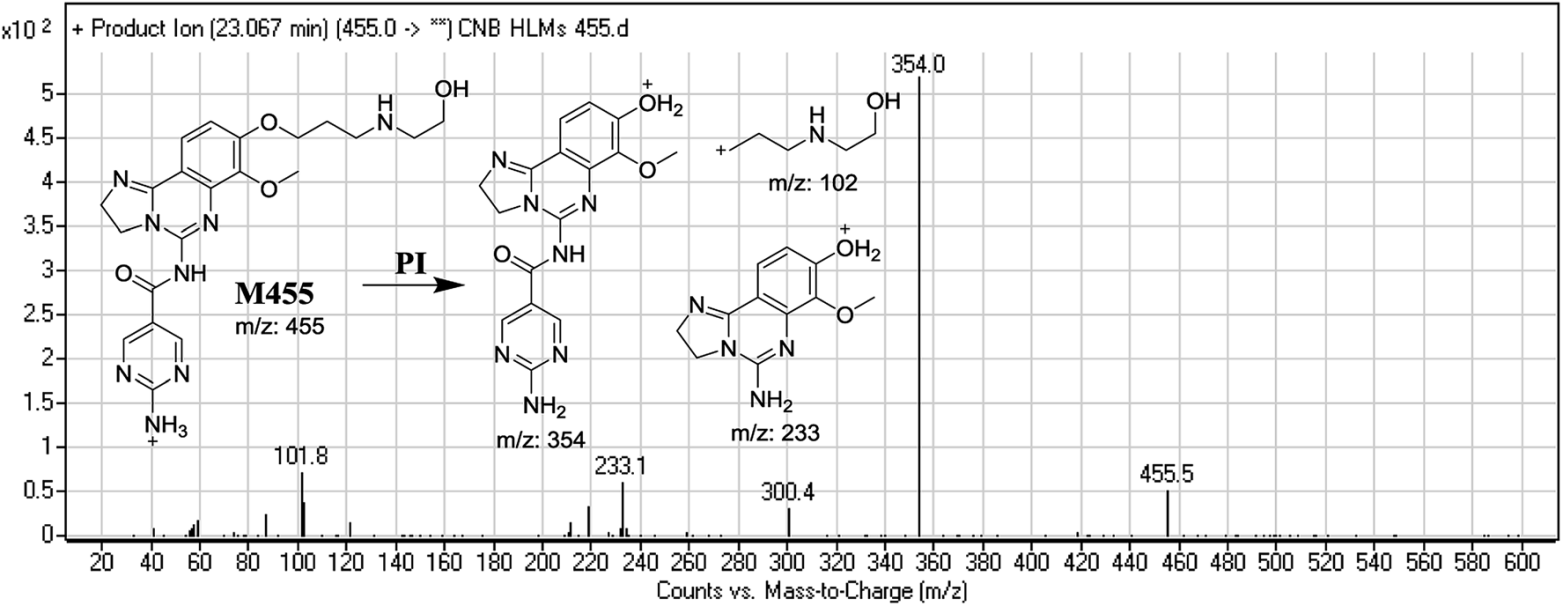
Mass spectrum of M455 and its MS/MS fragments.

M455 PIP appeared at 23.0 min. F of PI at *m*/*z* 455 resulted in three DRs at *m*/*z* 354, and *m*/*z* 233, *m*/*z* 102. DR at *m*/*z* 102 represented the cleaved morpholine ring and dealkylation of the ethyl moiety matching that of other ions at *m*/*z* 354 and *m*/*z* 233. Ring cleavage and dealkylation were predicted to occur at the morpholine ring (Fig. 5).

#### 3.2.4. Identification of M483a and M483b phase I metabolites of CNB

**Fig. 6:**
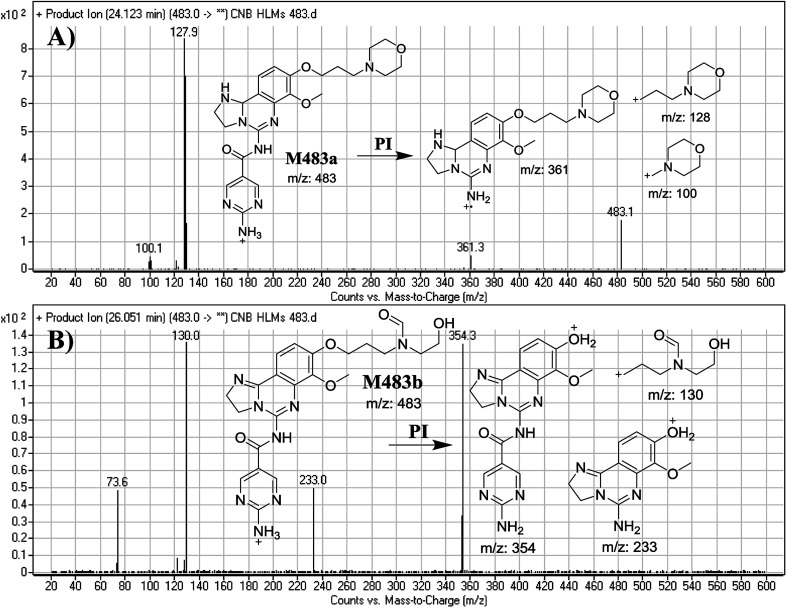
Mass spectra of M483a (A) and M483b (B) and their MS/MS fragments.

M483a and M483b PIPs appeared at 24.1 min and 26.1 min, respectively. F of PI at *m*/*z* 483 generated different DRs (Fig. 6A and B).

F of M483a resulted in three DRs at *m*/*z* 361, *m*/*z* 128 and *m*/*z* 100 (Fig. 6A), which represents the alkyl morpholine ring produced by single bond cleavage. DR at *m*/*z* 128 suggested that no metabolic change occurred at the morpholine ring. DRs revealed that M483a is a reduced form of CNB at the 2,3-dihydroimidazole ring (Fig. 6A).

F of M483b resulted in three DRs at *m*/*z* 354, *m*/*z* 233, and *m*/*z* 130 (Fig. 6B). DR at *m*/*z* 130 suggested that metabolic changes occurred in the morpholine ring matching the other DR at *m*/*z* 354. Ring cleavage and oxidative dealkylation were predicted to occur at the morpholine ring (Fig. 6B).

#### 3.2.5. Identification of M499 phase I metabolite of CNB

**Fig. 7:**
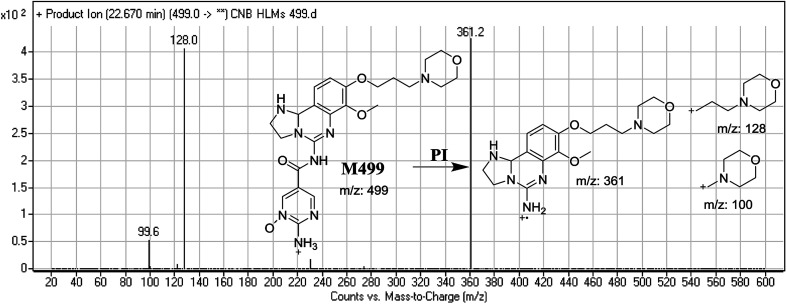
Mass spectrum of M499 and its MS/MS fragments.

M499 PIP appeared as [M + H]^+^ (*m*/*z* 499) at 22.7 min. F of PI at *m*/*z* 499 resulted in DRs at *m*/*z* 128 and *m*/*z* 100, indicating the absence of a metabolic reaction at the morpholine ring. DR at *m*/*z* 361 suggested that reduction occurred in the dihydroimidazole ring whereas *N*-oxidation occurred at the pyrimidine ring (Fig. 7).

### 3.3. Reactive metabolites

Two cyano and 2 methoxyl adducts were identified after incubating CNB with HLMs in the presence of trapping agents.

#### 3.3.1. Identification of M506 cyano conjugate of CNB

**Fig. 8:**
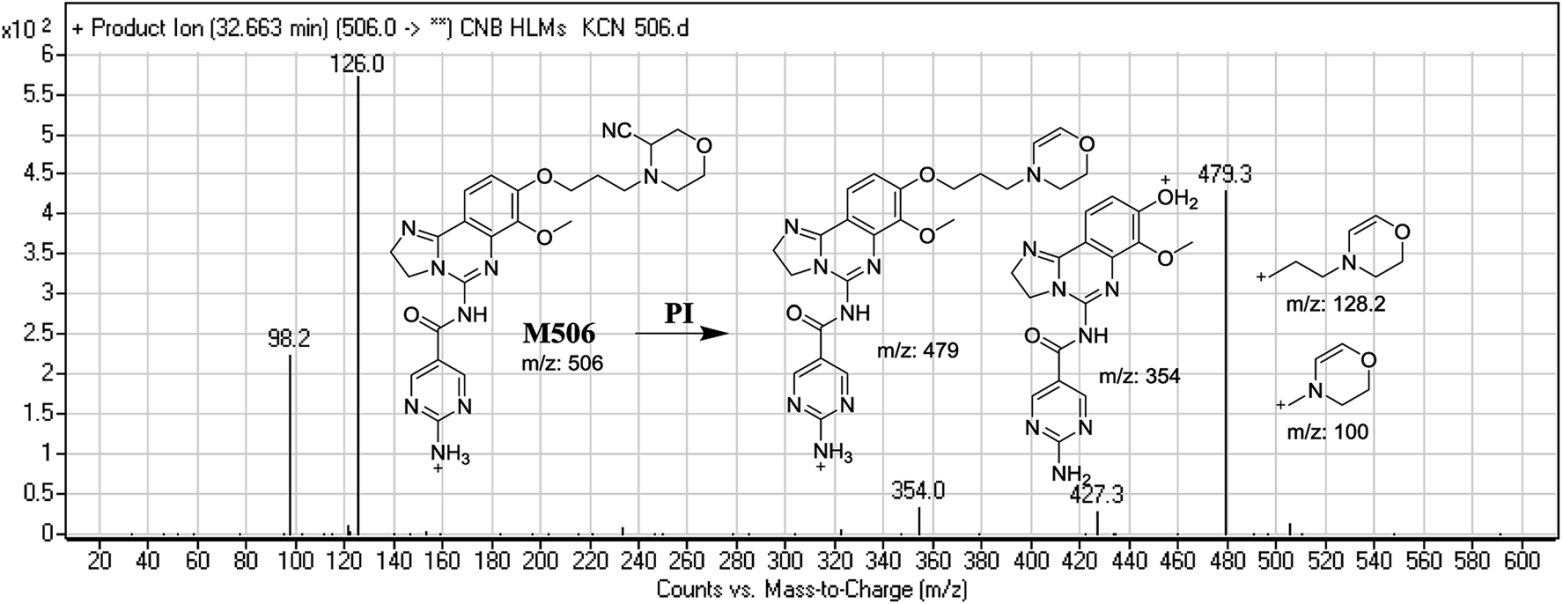
Mass spectrum of M506 and its MS/MS fragments.

M506 PIP appeared at 32.6 min. F of PI at *m*/*z* 506 resulted in DRs at *m*/*z* 479, *m*/*z* 354, *m*/*z* 126, and 98 *m*/*z*. DR at *m*/*z* 479 indicated the loss of a hydrogen cyanide group. DR at *m*/*z* 126 suggested the addition of a cyanide ion at the activated α-carbon of the N atom on the morpholine ring (Fig. 8).

#### 3.3.2. Identification of M508 cyano conjugate of CNB

**Fig. 9:**
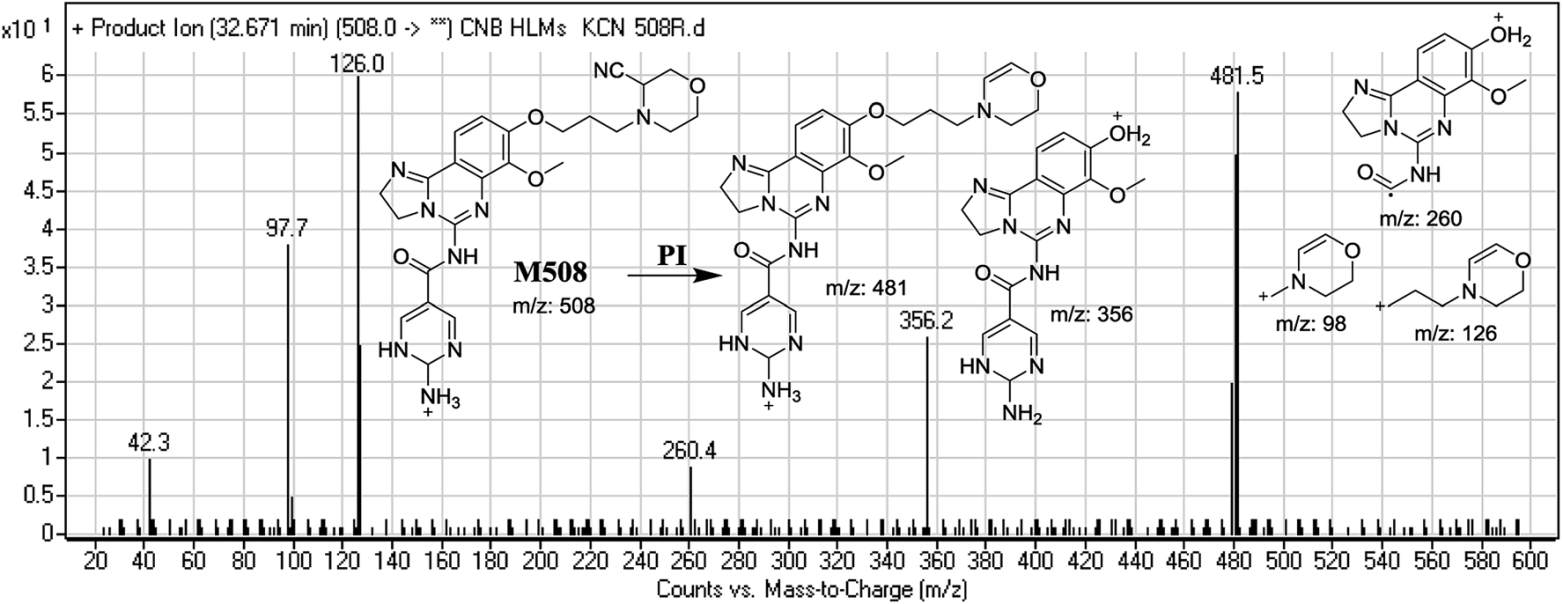
Mass spectrum of M508 and its MS/MS fragments.

M506 PIP appeared at 32.7 min. F of PI at *m*/*z* 508 resulted in DRs at *m*/*z* 481, *m*/*z* 356, *m*/*z* 126, and *m*/*z* 98. DR at *m*/*z* 481 indicated the loss of a hydrogen cyanide group*.* DRs at *m*/*z* 356 and *m*/*z* 260 suggested that a reduction occurred on the pyrimidine ring. DR at *m*/*z* 126 suggested that the addition of a cyanide ion occurred at the activated α-carbon of the N atom on the morpholine ring (Fig. 9).

#### 3.3.3. Identification of M439 methoxyamine conjugate of CNB

**Fig. 10:**
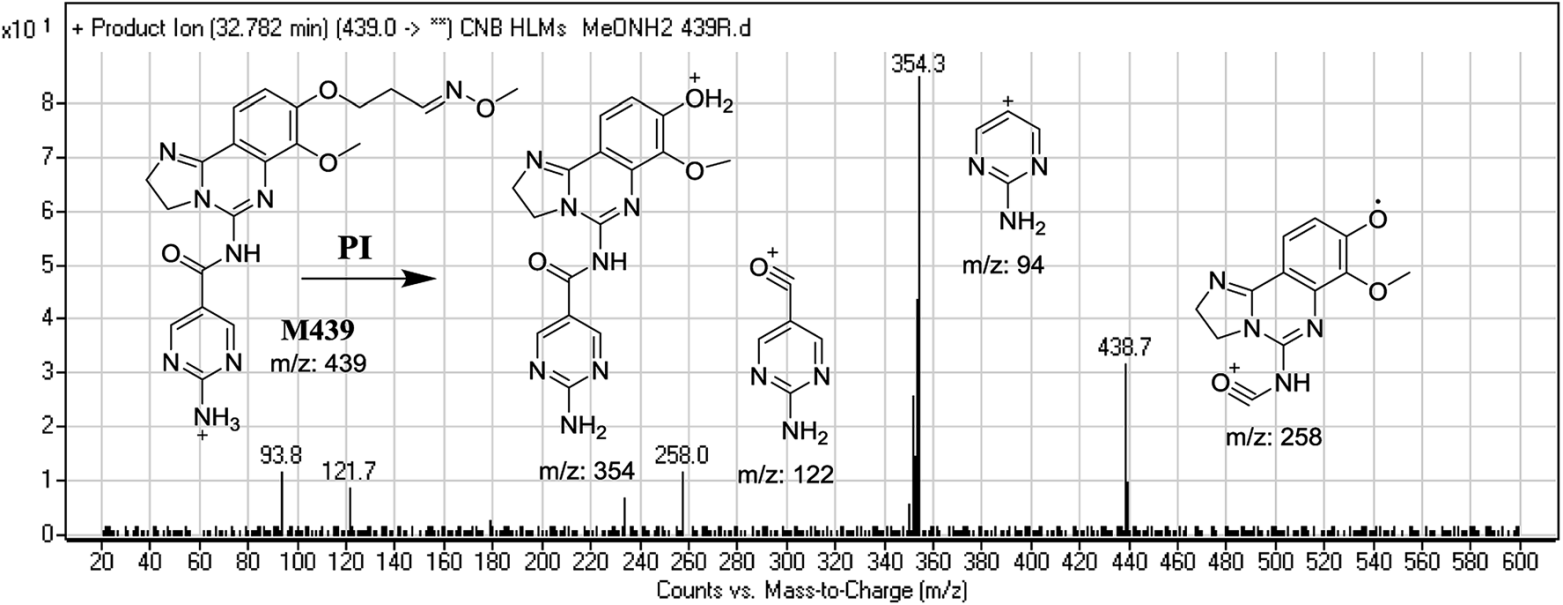
Mass spectrum of M439 at *m*/*z* 439 and its MS/MS fragments.

M439 PIP appeared as [M + H]^+^ (*m*/*z* 439) at 32.8 min. F of PI at *m*/*z* 439 resulted in DRs at *m*/*z* 354, *m*/*z* 258, *m*/*z* 122, and *m*/*z* 94. DR at *m*/*z* 354 indicated the formation of an oxime and suggested that all metabolic reactions occurred on the morpholine ring matching the three DRs (Fig. 10).

#### 3.3.4. Identification of M512 methoxyamine conjugate of CNB

**Fig. 11:**
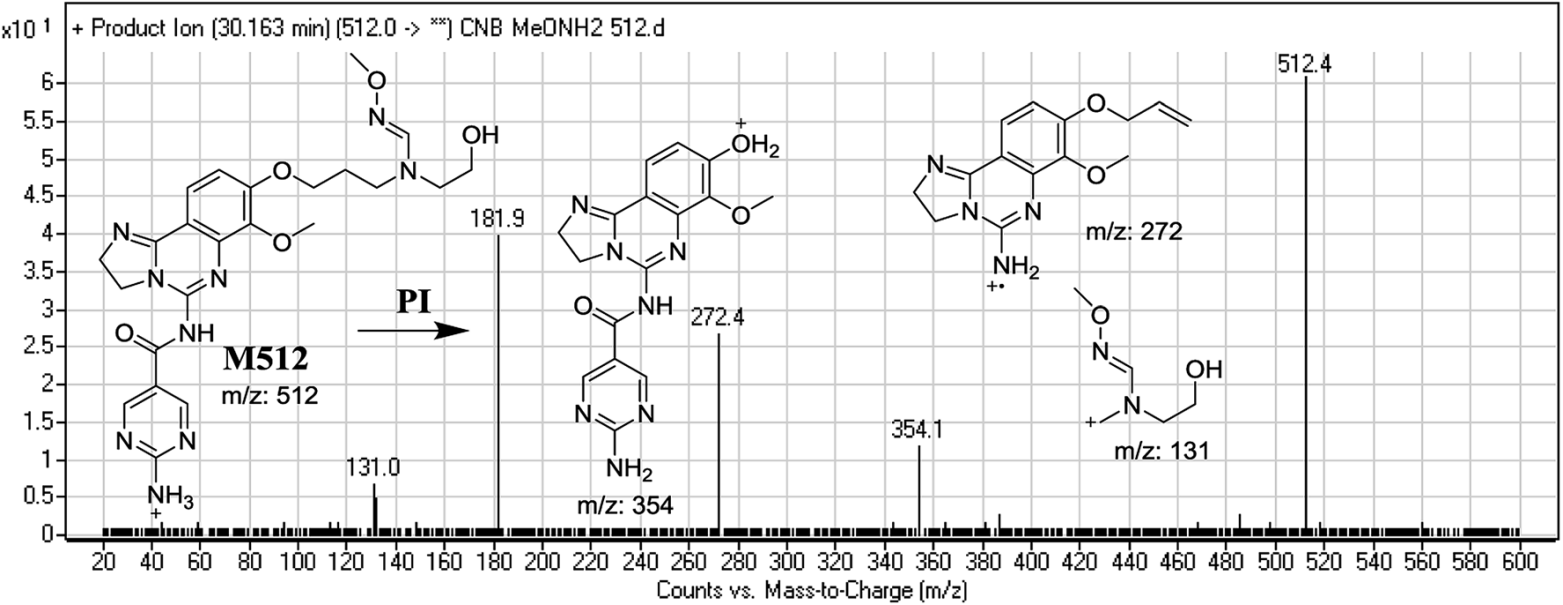
Mass spectrum of M512 at *m*/*z* 512 and its MS/MS fragments.

M512 PIP appeared as [M + H]^+^ (*m*/*z* 512) at 32.8 min. F of PI at *m*/*z* 512 resulted in DRs at *m*/*z* 354, *m*/*z* 272, and *m*/*z* 131. DR at *m*/*z* 354 suggested that an oxime was formed and all metabolic reactions occurred on the morpholine ring, which matched the other two DRs'. DR at *m*/*z* 131 indicated the dealkylation and oxidative opening of the morpholine ring, resulting in oxime with an aldehyde (Fig. 11).

### 3.4. Mechanism of CNB bioactivation

**Scheme 1:**
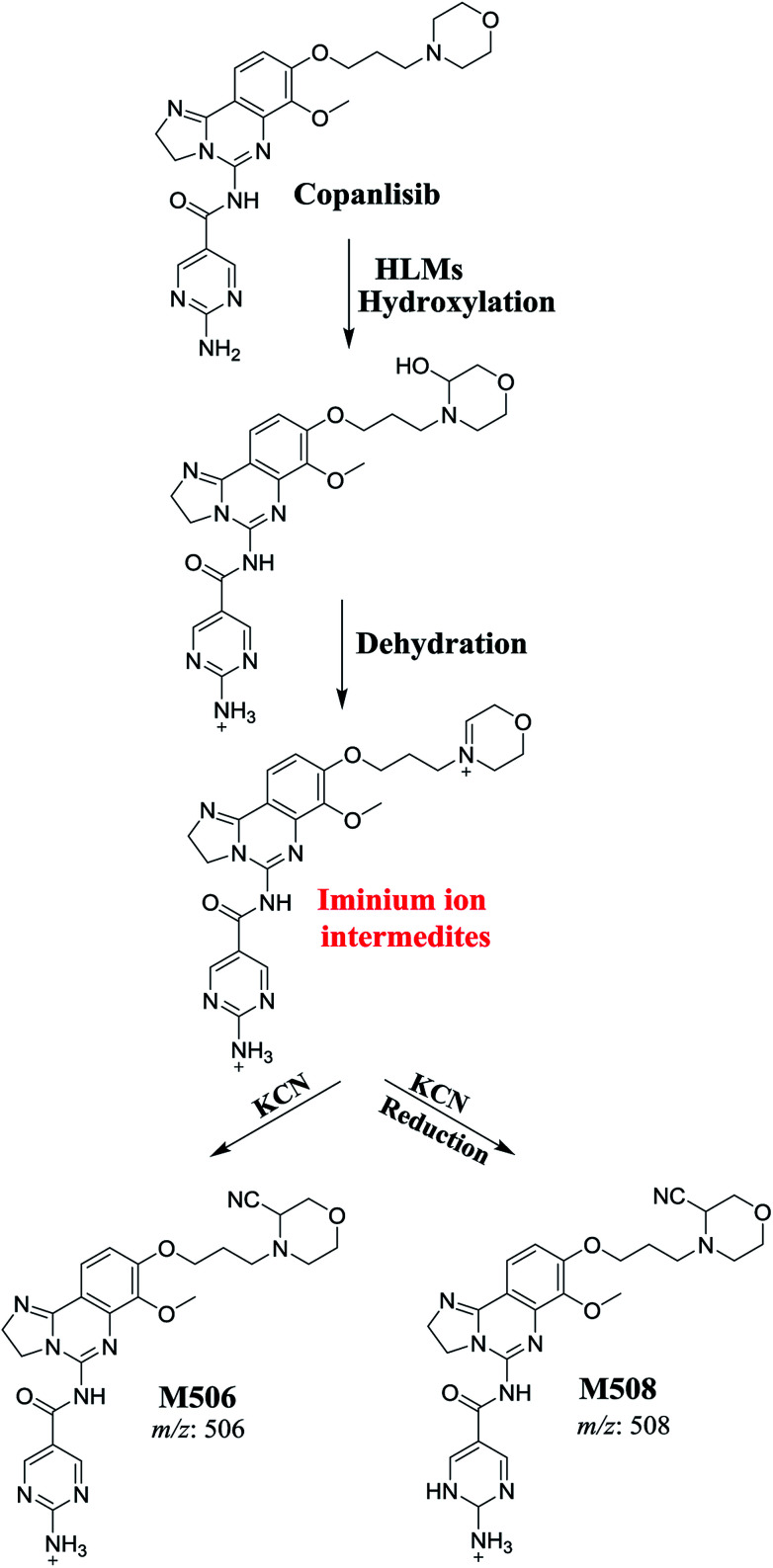
Proposed mechanism for the formation of iminium intermediates in CNB metabolism and the potential trapping strategy.

The generation of M506 and M508 cyanide adducts confirmed the formation of iminium intermediates, and hydroxylation of the piperazine ring in CNB followed by the loss of a water molecule created unstable iminium electrophiles that could be captured by a cyanide nucleophile to form a stable adduct (Scheme 1). The mechanism of the formation of the iminium intermediate and CNB bioactivation have been previously studied in cyclic tertiary amine-containing drugs.^33–38^

**Scheme 2:**
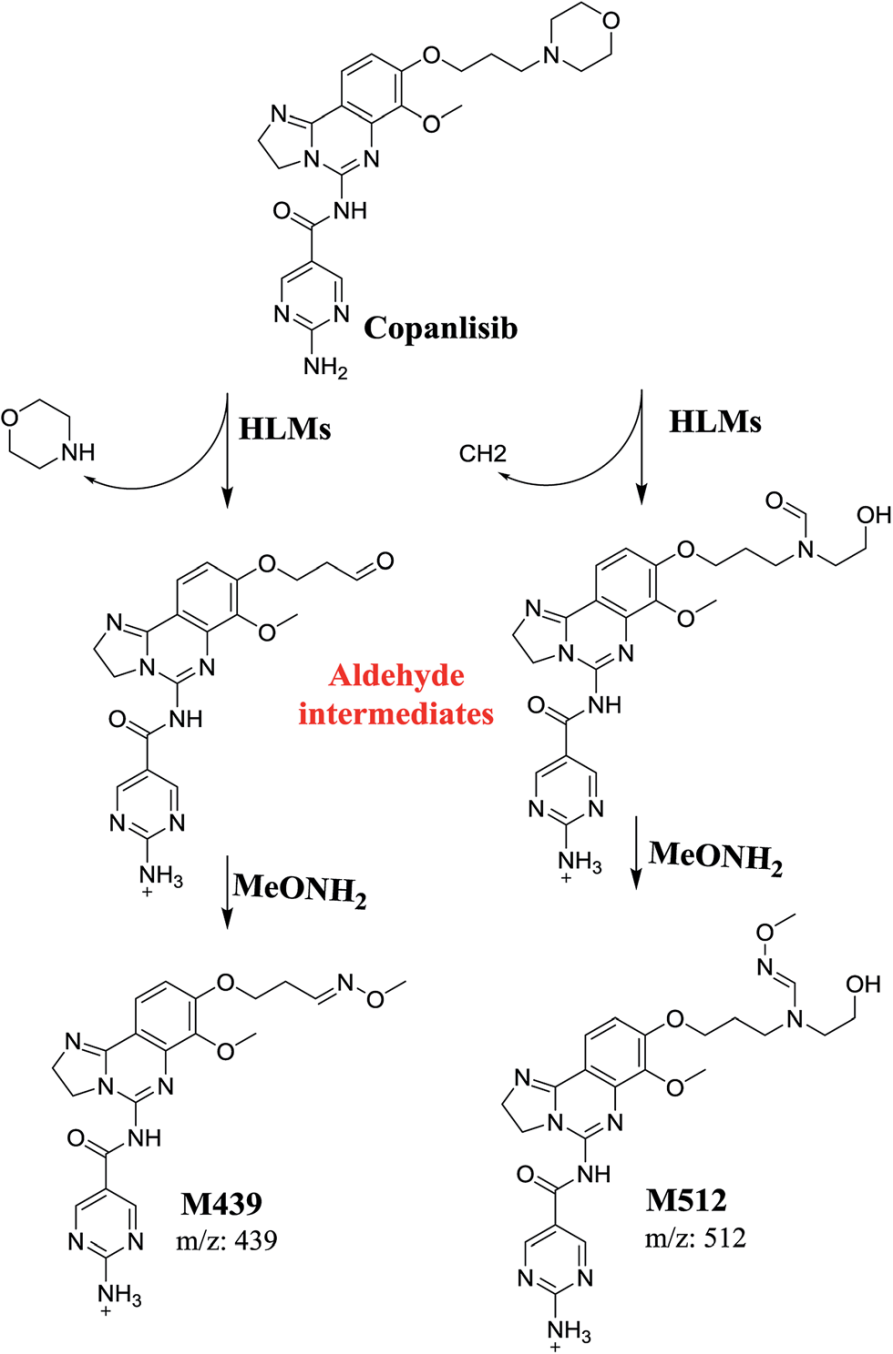
Proposed mechanism of aldehyde generation.

The formation of M439 and M512 confirmed the production of aldehyde intermediates in CNB metabolism. The aldehyde electrophiles were formed by oxidative dealkylation and captured with methoxyamine-forming oxime (M439 and M512). Oxidative dealkylation of the morpholine group formed an aldehyde captured by methoxyamine-forming M439. The opening of the morpholine ring by oxidative dealkylation resulted in aldehyde entrapment *via* M512 formation. Both oximes were stable, and were identified using LC-MS/MS (Scheme 2). Aldehyde formation in morpholine group-containing drugs has been described previously.^39,40^

## 4. Conclusions

**Fig. 12:**
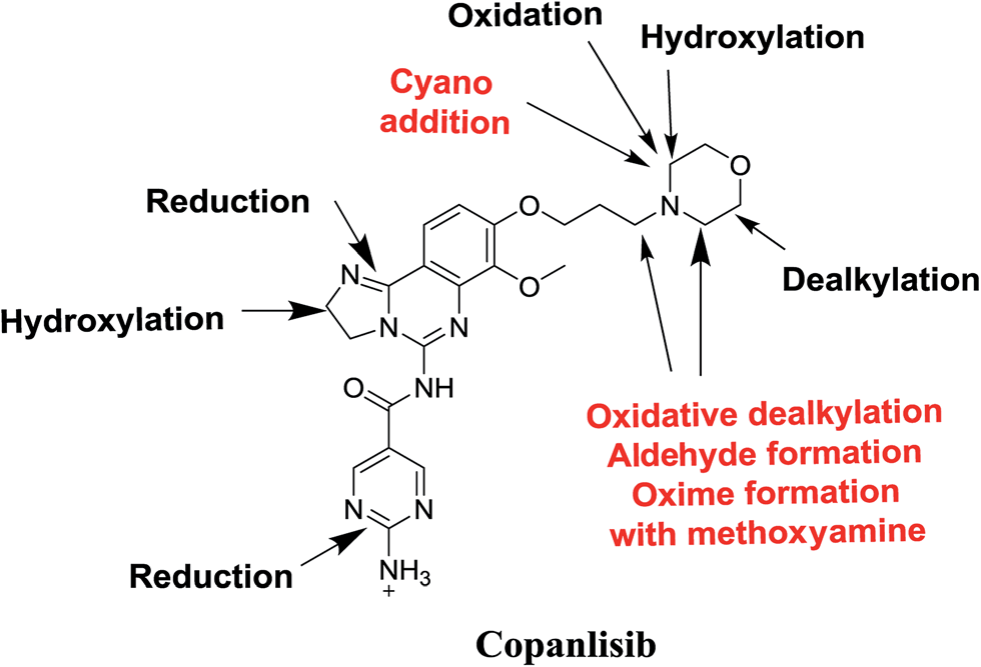
Chemical structure of copanlisib. Sites of phase I metabolic reactions and pathways of bioactivation are indicated by arrows.

Seven phase I metabolites, 2 cyano adducts, and 2 methoxyamine adducts of CNB were detected (Fig. 12). All pathways for the reactive metabolites depended on the morpholine group of CNB, which may be associated with the side effects caused by CNB. These results have provided relevant groundwork for further investigations on CNB toxicity. Profiling of phase I metabolites is very crucial as it may be the next generation drugs. Understanding the bioactivation pathways is crucial to identify bioactive soft spots. Isosteric replacement or steric hindrance groups at these locations may block bioactivation and retain the pharmacological activity. Therefore, using the aforementioned data will help to develop next-generation drugs with less adverse effects.

## Conflicts of interest

The authors declare no conflict of interest.

## Supplementary Material

RA-009-C8RA10322D-s001
