# Supplementary material for: Reactive intermediates in copanlisib metabolism identified by LC-MS/MS: phase I metabolic profiling
Source: RSC Adv. 2019 Feb 21;9(11):6409–18. doi: 10.1039/c8ra10322d (PMC9060959; doi:10.1039/c8ra10322d)
Supplement: RA-009-C8RA10322D-s001 [file RA-009-C8RA10322D-s001.pdf]

Electronic Supplementary Material (ESI) for RSC Advances.
This journal is © The Royal Society of Chemistry 2019

Supplementary data

1- *In vitro* phase I metabolites of copanlisib.

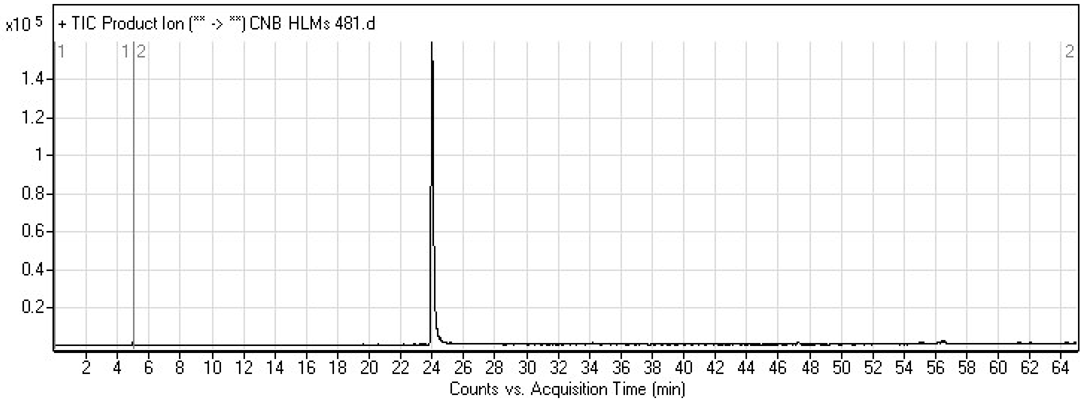

Fig. S1: PI chromatogram of PIP at *m/z* 481 showing CNB peak at 24.4 min.

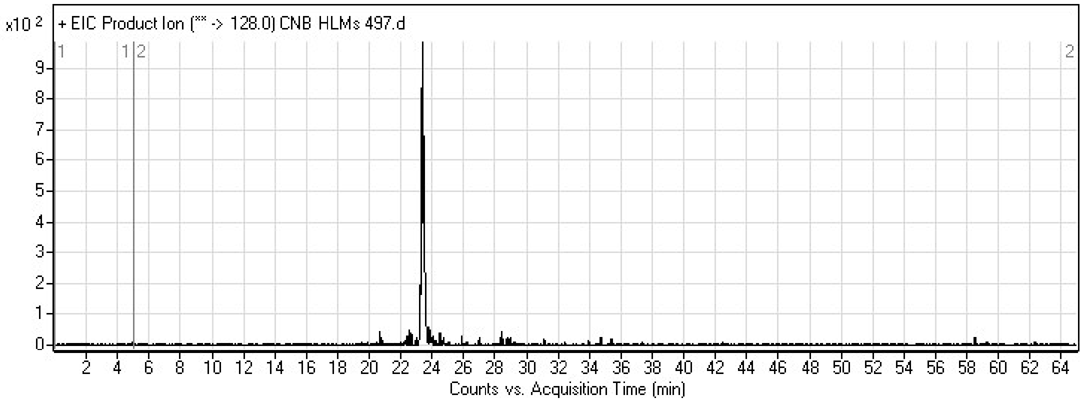

Fig. S2: PI chromatogram of CNB497a and CNB497b peaks at 23.4 min and 23.8, respectively,

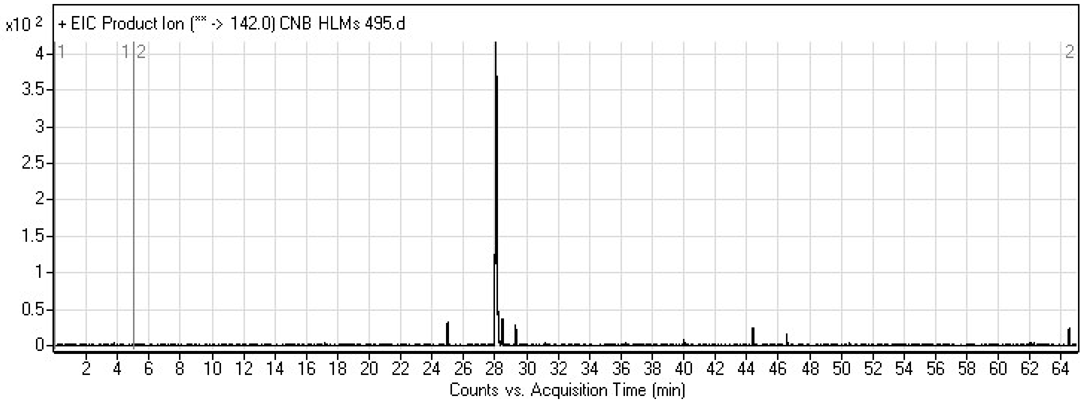

Fig. S3: PI chromatogram of CNB495 peak at 28.0 min.

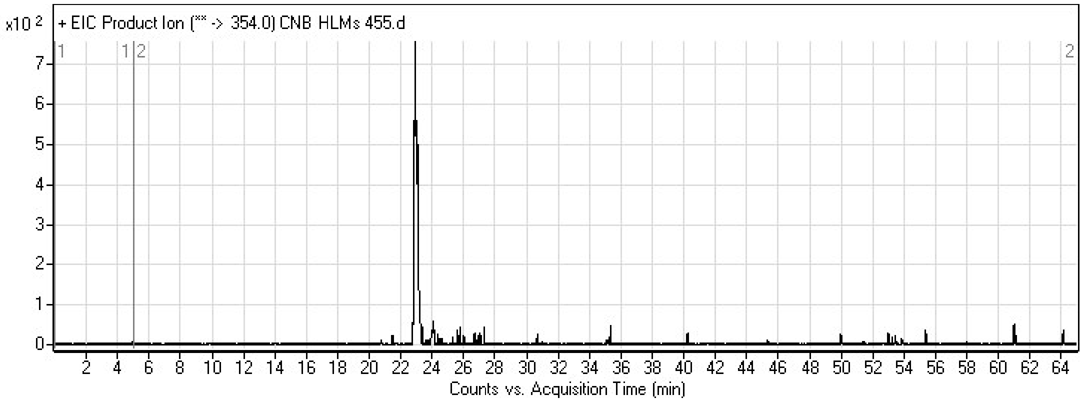

Fig. S4: PI chromatogram of CNB455 peak at 23.0 min.

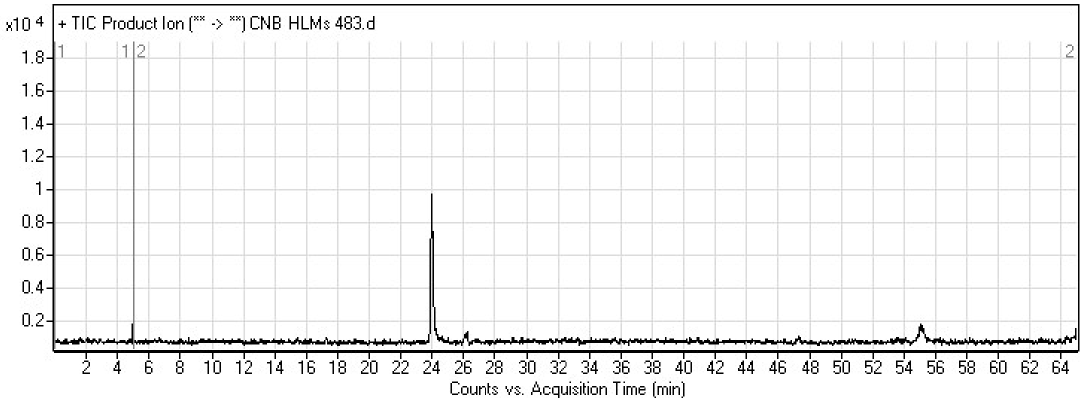

Fig. S5: PI chromatogram of CNB483a and CNB483b peaks at 24.1 min and 26.1 min, respectively (A).

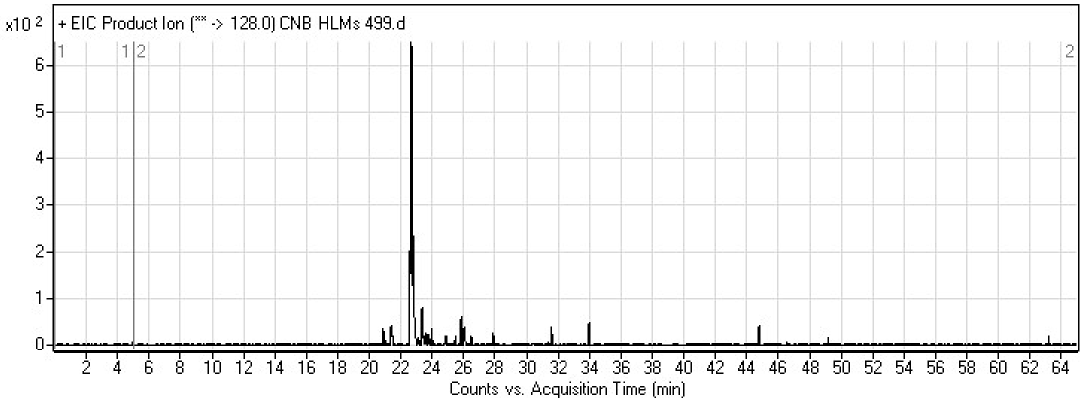

Fig. S6: PI chromatogram of PIP CNB499 peak at 22.6 min.

2- Reactive intermediates of copanlisib:

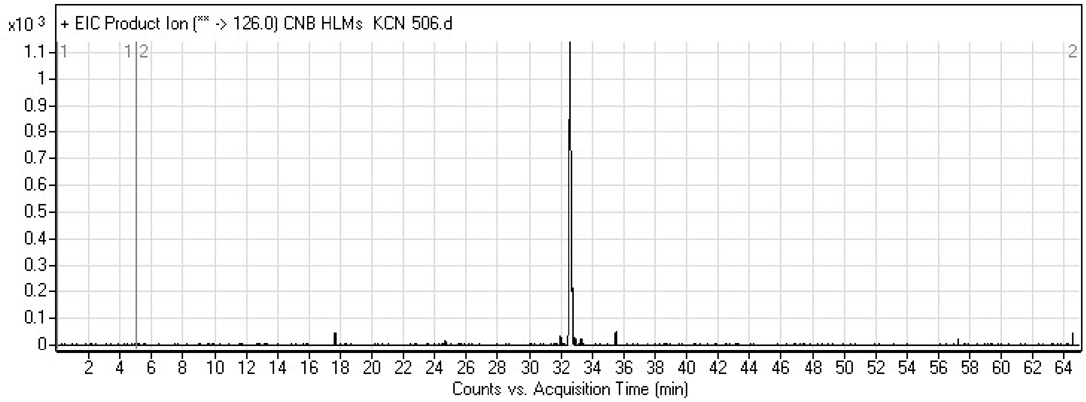

Fig. S7: PI chromatogram of CNB506 peak at 32.6 min (A).

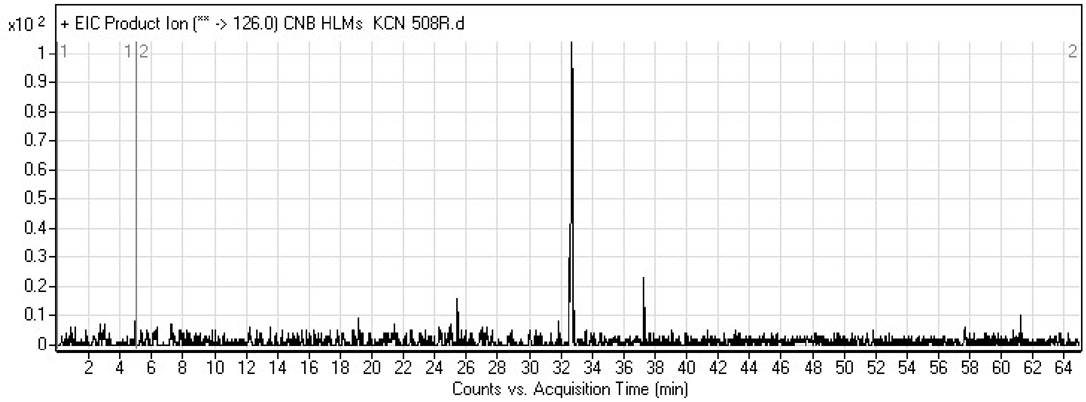

Fig. S8: PI chromatogram of CNB508 peak at 32.7 min.

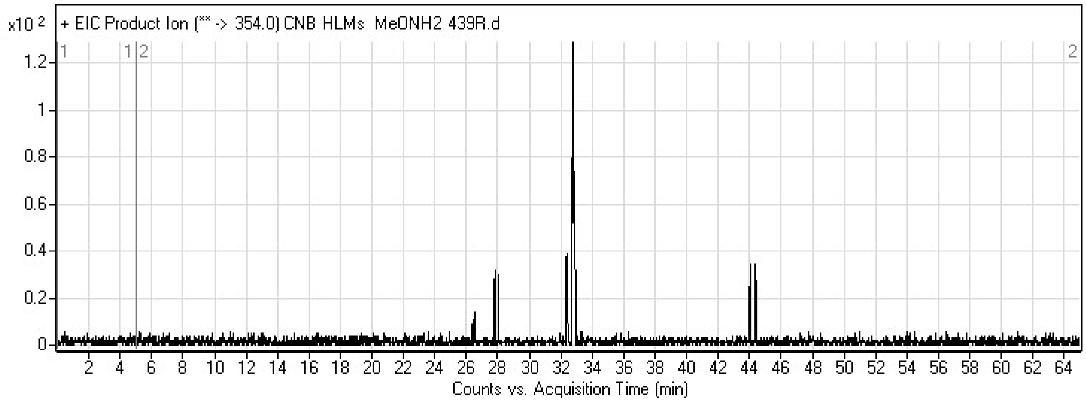

Fig. S9: PI chromatogram of CNB439 peak at 32.8 min.

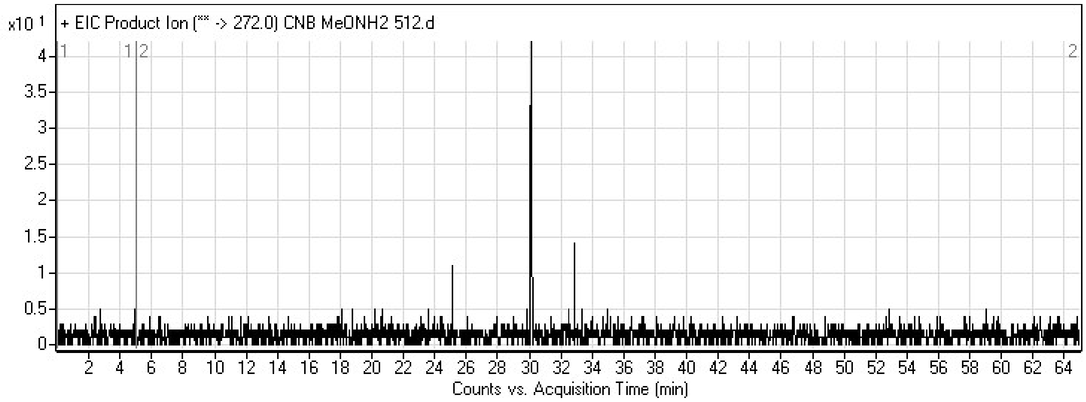

Fig. S10: PI chromatogram of CNB512 peak at 30.2 min.

3- Control chromatograms of copanlisib.

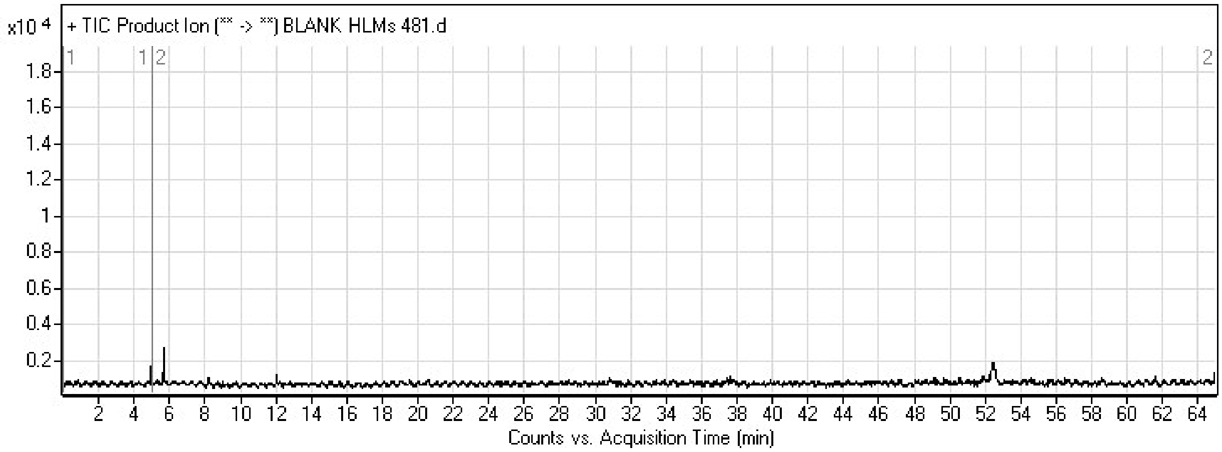

Fig. S11: PI chromatogram of PIP at *m/z* 481 showing the absence of CNB peak at 24.4 min.

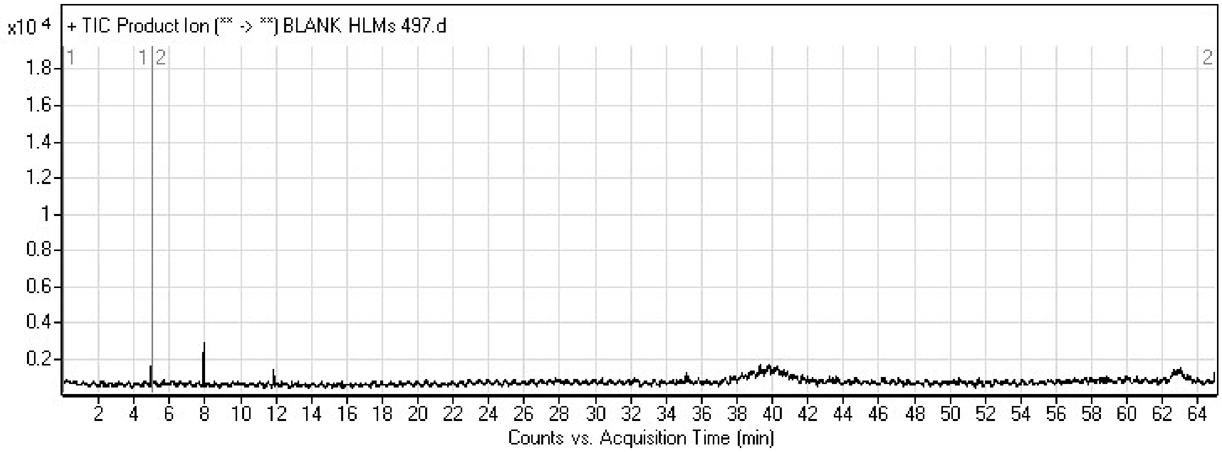

Fig. S12: PI chromatogram showing the absence of CNB497a and CNB497b peaks at 23.4 min and 23.8, respectively,

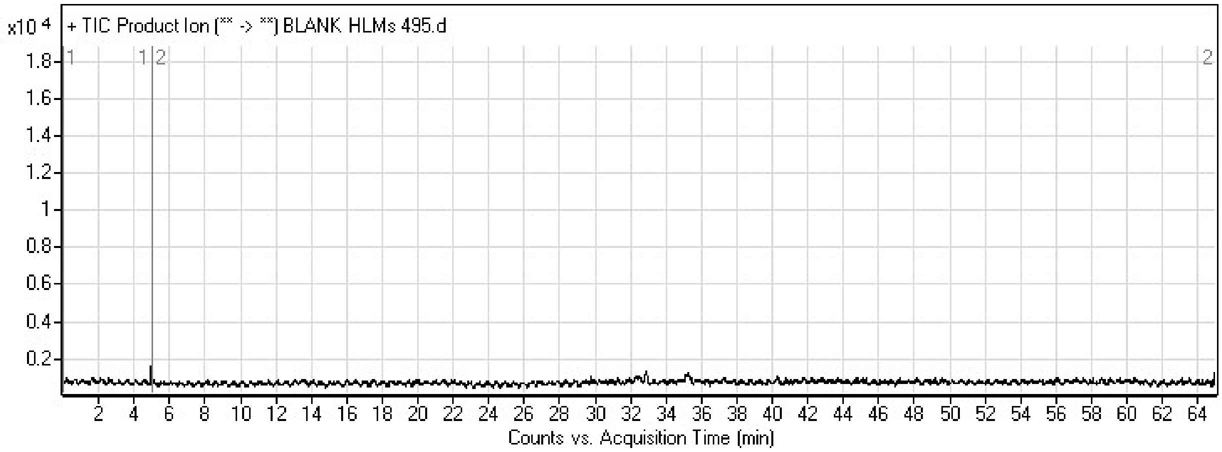

Fig. S13: PI chromatogram showing the absence of CNB495 peak at 28.0 min.

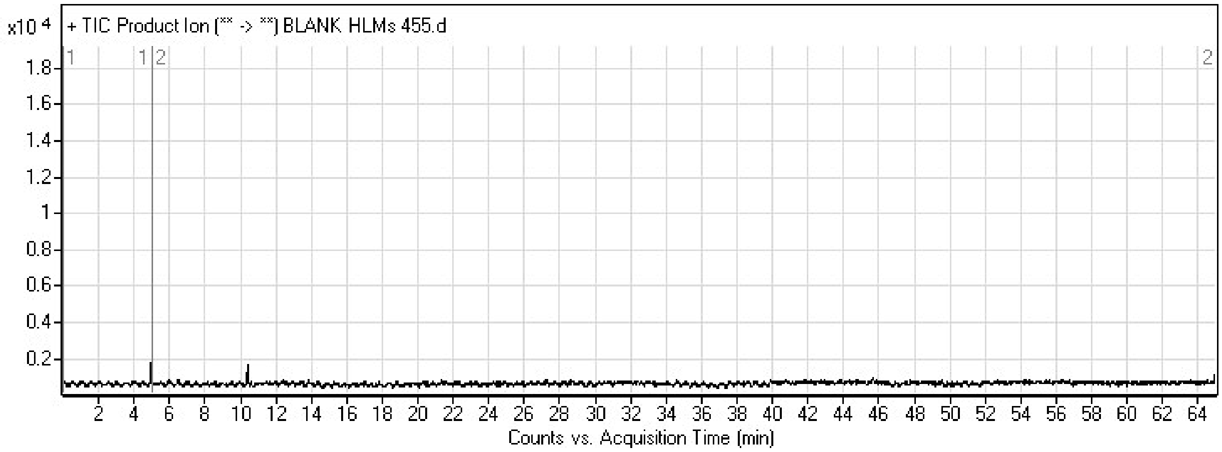

Fig. S14: PI chromatogram showing the absence of CNB455 peak at 23.0 min.

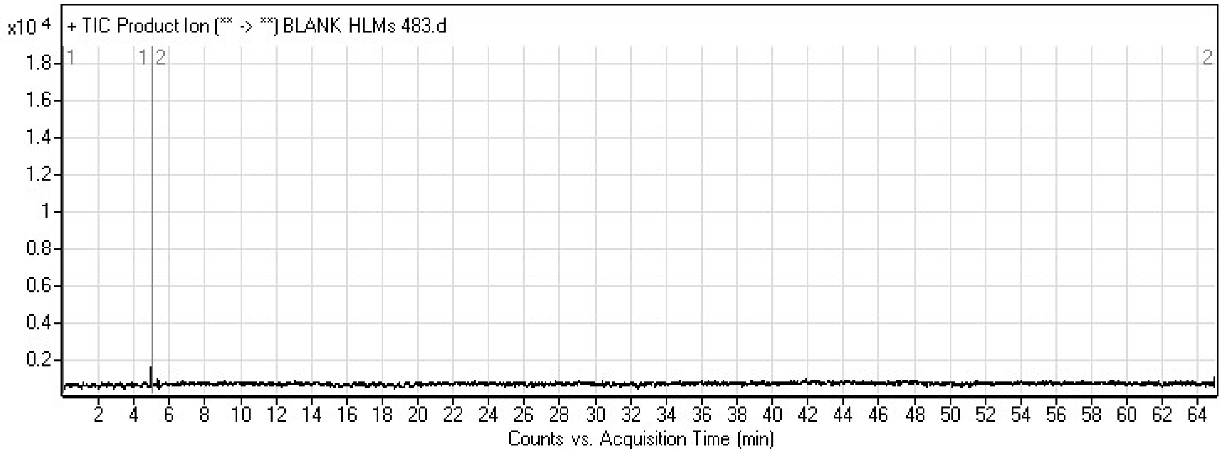

Fig. S15: PI chromatogram showing the absence of CNB483a and CNB483b peaks at 24.1 min and 26.1 min, respectively (A).

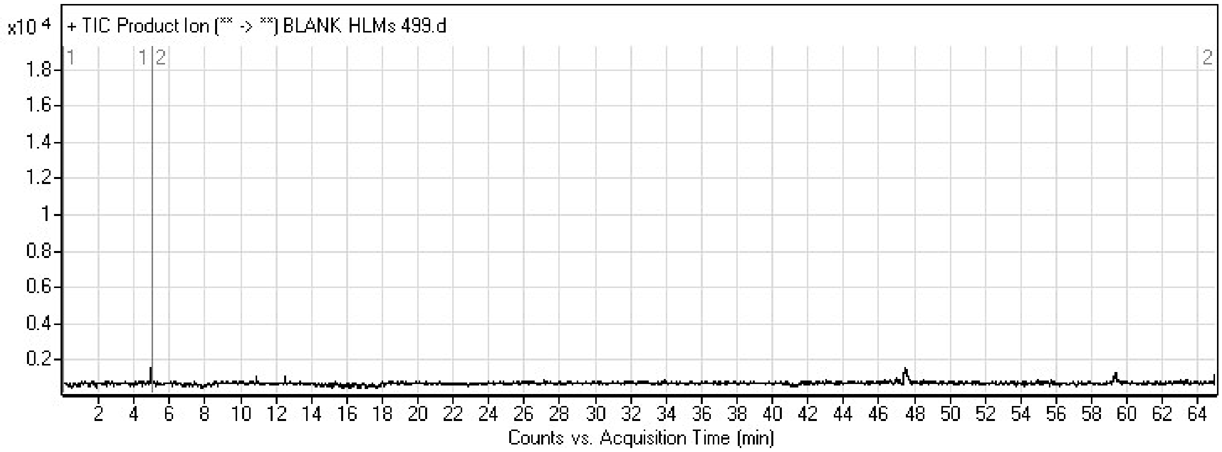

Fig. S16: PI chromatogram showing the absence of PIP CNB499 peak at 22.6 min.

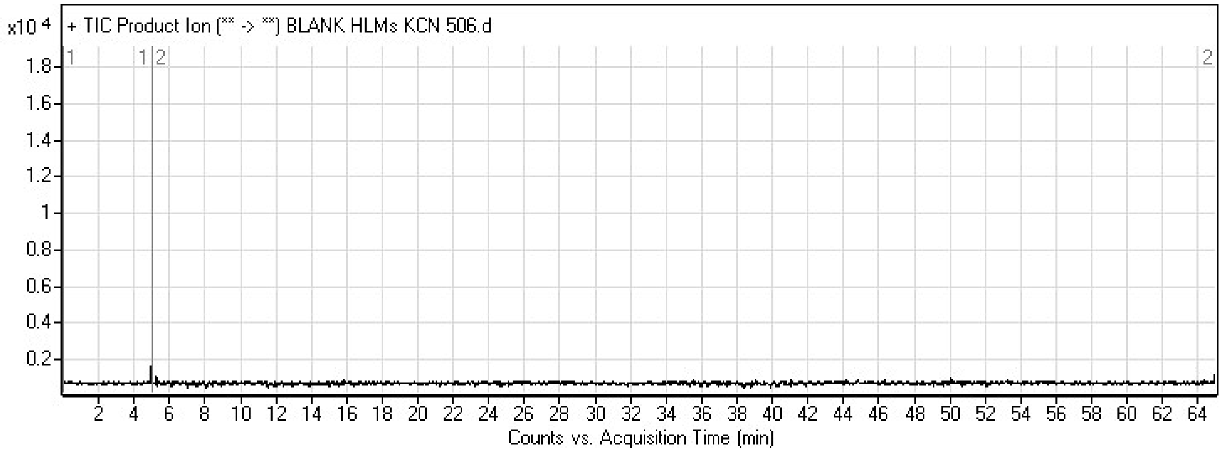

Fig. S17: PI chromatogram showing the absence of CNB506 peak at 32.6 min (A).

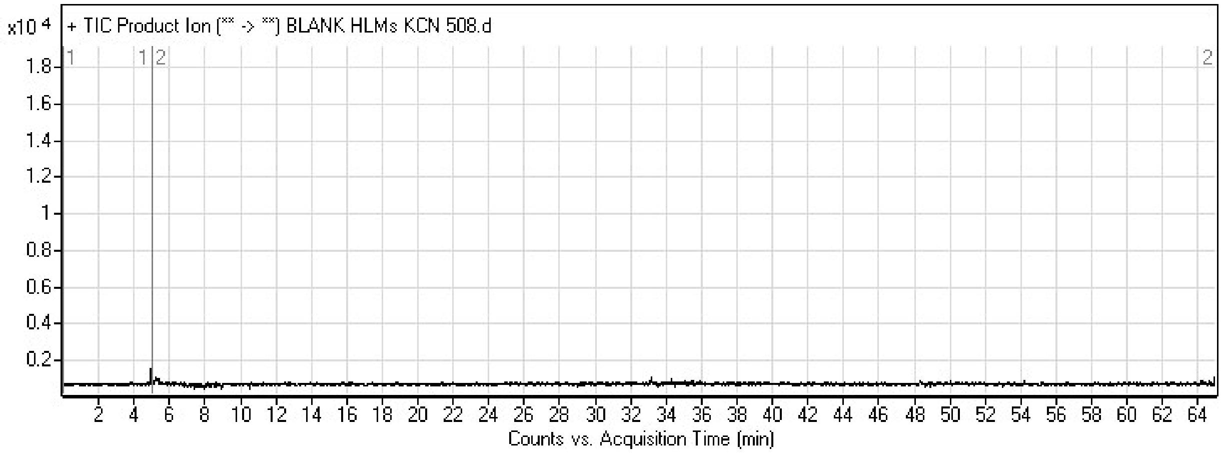

Fig. S18: PI chromatogram showing the absence of CNB508 peak at 32.7 min.

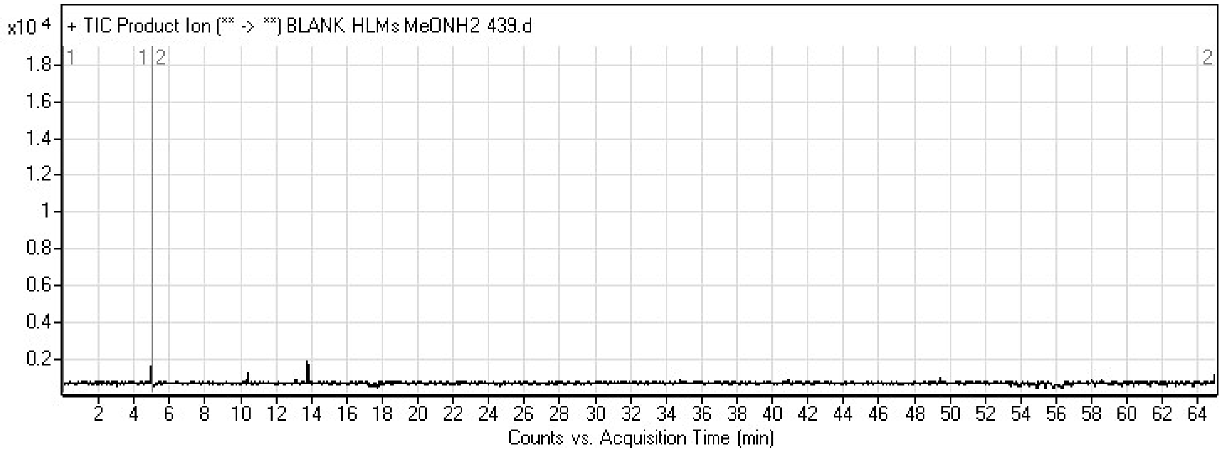

Fig. S19: PI chromatogram showing the absence of CNB439 peak at 32.8 min.

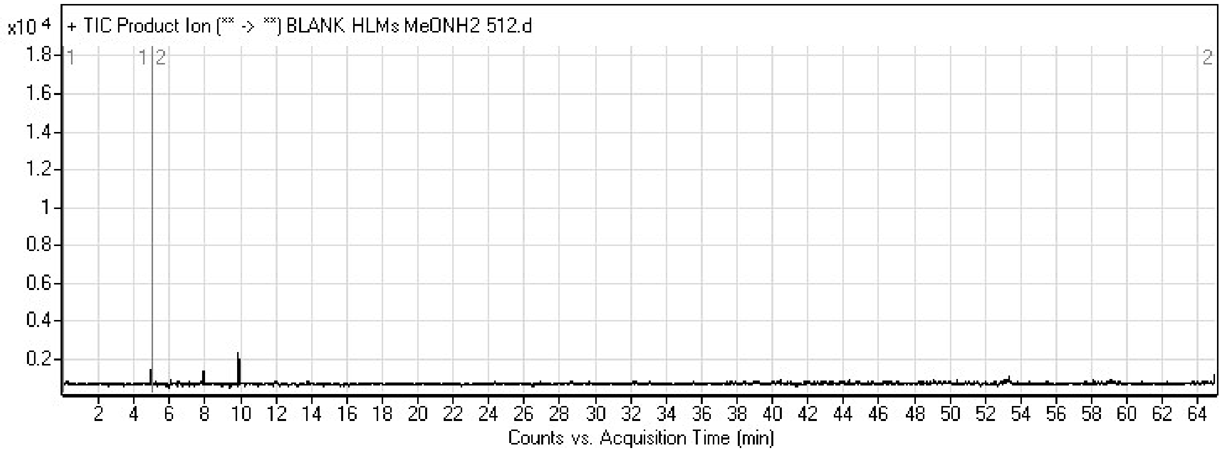

Fig. S20: PI chromatogram showing the absence of CNB512 peak at 30.2 min.
